# Crossword puzzles: self-learning tool in pharmacology

**DOI:** 10.1007/s40037-012-0033-0

**Published:** 2012-11-09

**Authors:** Nitin Gaikwad, Suresh Tankhiwale

**Affiliations:** 1 Department of Pharmacology, Jawaharlal Nehru Medical College, Datta Meghe Institute of Medical Sciences (Deemed University), Sawangi (Meghe), Wardha, 442004 Maharashtra India; 2 Department of Health Professions Education, Jawaharlal Nehru Medical College, Datta Meghe Institute of Medical Sciences (Deemed University), Sawangi (Meghe), Wardha, 442004 Maharashtra India; 3 ‘Safal’ 242, Datta Nagar, Near Shastri Nagar High School, Old Bagadganj Road, Nagpur, 440008 Maharashtra India

**Keywords:** Crossword puzzles, Self-learning, Active learning, Pharmacology, Recreational learning

## Abstract

Students of the second professional MBBS course of the Indian medical curriculum (II MBBS) perceive pharmacology as a ‘Volatile Subject’ because they often find it difficult to remember and recall drug names. We evaluated the usefulness of crossword puzzles as a self-learning tool to help pharmacology students to remember drug names. We also measured the students’ satisfaction with this learning method. This was an open-label randomized, two-arm intervention study, conducted with II MBBS students (n = 70), randomly selected and assigned to two groups A (n = 35) and B (n = 35). Two self-learning modules containing crossword puzzles with antihypertensive and antiepileptic drug terms were prepared and pre-validated. Hard copies of both crossword puzzles were administered to Group A (Intervention group) on two different occasions. One hour was allotted to solve a puzzle. Students were allowed to refer to their textbooks. Group B (Control group) underwent the self-learning module without the crossword puzzles. In both groups, pre- and post-test knowledge was assessed. Students’ perceptions of the crossword puzzles were assessed using a pre-validated 10-item questionnaire. Responses to items 1–8 were recorded using a 5-point Likert scale. Responses to item 9 were recorded on a 10-point rating scale while item 10 was an open–ended question. The crossword completion index was 92.86 %. In group A, the average pre-test score was 6.09 whereas the average post-test score was 12.87 (*p* < 0.05). In group B, average pre- and post-test scores were 6.03 and 9.74, respectively. A significant difference (*p* < 0.001) was observed between the post-test scores of the two groups. The absolute learning gain was 33.9 % in Group A and 18.55 % in Group B. The response rate for the questionnaire was 100 %. Of the students, 71.43 % strongly agreed that crossword puzzles enhanced their knowledge of antihypertensive and antiepileptic drugs and were helpful for remembering and recalling the drug names, 60 % students found it challenging and a good problem solving activity and 85.71 % students opined that it was a good self-learning, recreational activity. The test scores improved when crossword puzzles, designed to improve retention of drug names, were incorporated in the self-study modules of pharmacology training. Students rated the crossword puzzles as a challenging and effective self-learning tool. Students’ acceptability for crossword puzzles further favours their usefulness as a self-learning tool. The crossword puzzle is an effective tool for ‘recreational learning’ and can be used as an active learning strategy to promote self-directed learning.

## Introduction

Teaching–learning methods are broadly classified as teacher controlled and learner controlled methods [1]. Among the various learner controlled methods, self-learning methods have their own importance and relevance. Various studies in medical education have shown that self-learning methods should be incorporated into the teaching–learning process to make learning more effective, efficient and meaningful.

Self-learning promotes active learning and critical thinking which in turn enhances self-reliance and in this process teachers can manage their time effectively to reinforce knowledge and skills. Moreover, for students, learning on their own can be an enjoyable experience [2].

Active learning strategies have their own benefits which primarily include fostering development of critical thinking, communication, cooperative learning skills and attitudes and values; promoting concept formation; providing an avenue for discovering misconceptions; and increasing motivation [3]. Various formats of interactive games [4–9] and puzzles [3, 10–12] have been advocated by researchers as active learning strategies which create an interactive learning experience by transforming inactive learning material into learning episodes where the learners are active players and participants.

Crossword puzzles are commonly found in newspapers and magazines. Crossword puzzles have also appeared in medical and nursing journals [12, 13] to review and summarize information in an engaging manner.

However, they have not yet been well explored as a self-learning tool in pharmacology. Pharmacology is a subject in the Second Professional MBBS course of the Indian medical curriculum (II MBBS). During the II MBBS course in pharmacology, students get acquainted with the core areas of this subject such as drug classification, pharmacokinetic and pharmacodynamic principles of drugs, therapeutic uses, adverse effects and contraindications of drugs.

The students often perceive pharmacology as a ‘Volatile Subject’. Students are introduced to newer terms and concepts in pharmacology and it has been observed that students often find it difficult to remember and recall drug names. Hence, reinforcement of key concepts is essential to increase understanding, learning and retention.

Crossword puzzles stimulate the mind, increase the vocabulary and help to develop healthy scepticism [3]. Therefore, instead of passive memorization of material, crossword puzzles can be beneficial as a self-learning tool to promote active learning and to develop critical thinking. Hence, the present study was conducted with the following objectives:

- to evaluate the usefulness of crossword puzzles as a self-learning tool in pharmacology;
- to evaluate the perceptions of students about crossword puzzles as a self-learning tool.

## Materials and methods

### Ethics committee approval

The study was conducted after obtaining approval from the Institutional Ethics Committee.

### Study design and sample

This was an open-label, randomized, controlled, parallel group intervention study conducted with 5th semester students of the second professional MBBS course (II MBBS students in the exam term). Students were selected using systematic random sampling. Every second roll number from the student attendance list was selected. The total sample size was 70.

### Study material

#### Crossword puzzles

We conducted an informal discussion with the pharmacology faculties and students to decide which topics would be included in the crossword puzzle activity. Faculties and students both suggested that the topics should be based on drugs for common ailments, should be from the ‘Must know’ portion of the pharmacology syllabus, and should have already been taught by traditional didactic teaching. Based on these suggestions, two self-learning modules containing crossword puzzles with antihypertensive and antiepileptic drug terms were prepared. Modules of 1-day duration were designed. Two didactic lectures of 1 h each had already been given on antihypertensive drugs and antiepileptic drugs.

The crossword puzzle clues (Across and Down) were verified from the standard textbooks of pharmacology. Before introducing the crossword puzzles to students, experts from the Pharmacology Department validated the content of the two crossword puzzles.

#### Pre-test and post-test

Pre- and post-tests for both the modules were prepared. Tests comprised multiple choice questions and short answer questions. The same set of questions was used for the pre- and post-test. The key to the questions was verified from the standard textbooks of pharmacology. The highest possible test score was 20. The pre- and post-tests were pre-validated.

#### Students’ feedback questionnaire

A questionnaire consisting of 10 items was prepared to record students’ perceptions about crossword puzzles as a self-learning tool in pharmacology. Items 1–9 were close–ended questions. The responses to items 1–8 were recorded on a 5-point Likert scale (1 = strongly disagree to 5 = strongly agree) while responses for item 9 were recorded on a 10-point scale (1 = not useful to 10 = very useful). Item 10 was an open–ended question and students were asked to give their comments.

#### Crossword activity

**Fig. 1:**
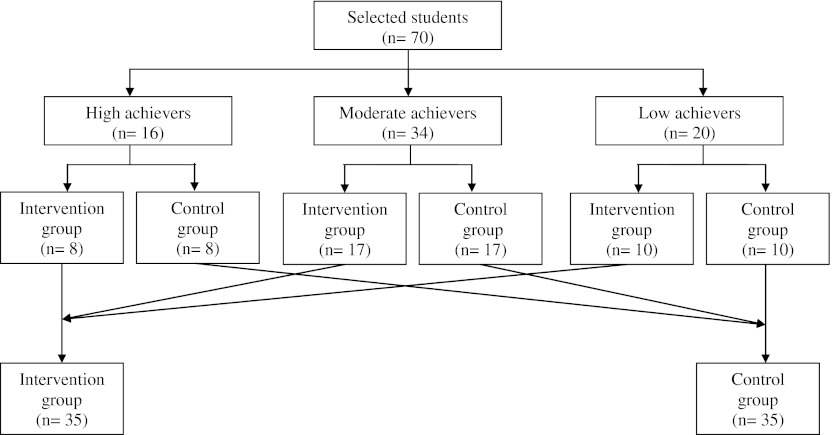
Flow of participants through the trial

The selected 70 students were further divided into high achievers (16), moderate achievers (34) and low achievers (20), based on their performance in first part of the completion test in pharmacology. According to our university criteria, we defined high achievers as students who score >75 %, moderate achievers as those with a score between 40 and 74.9 % and low achievers with a score <40 %. The students from these subgroups were then randomly assigned to two groups, Group A (n = 35) and Group B (n = 35) using a simple lottery method (Fig. 1). Thus, the two groups comprised an approximately equal number of high, moderate and low achievers, making them similar prior to intervention. These students were briefed about the study and the objectives of the study were explained. Written informed consent was taken from the selected students before their participation in the study.

Group A was an intervention group and Group B served as a control group. Printed copies of the crossword puzzles were administered to the students of Group A (Intervention group). One hour was allotted to solve the puzzle. Students solved the crossword puzzle individually. They were allowed to refer to textbooks and encouraged to use self-learning while solving the puzzles.

**Fig. 2:**
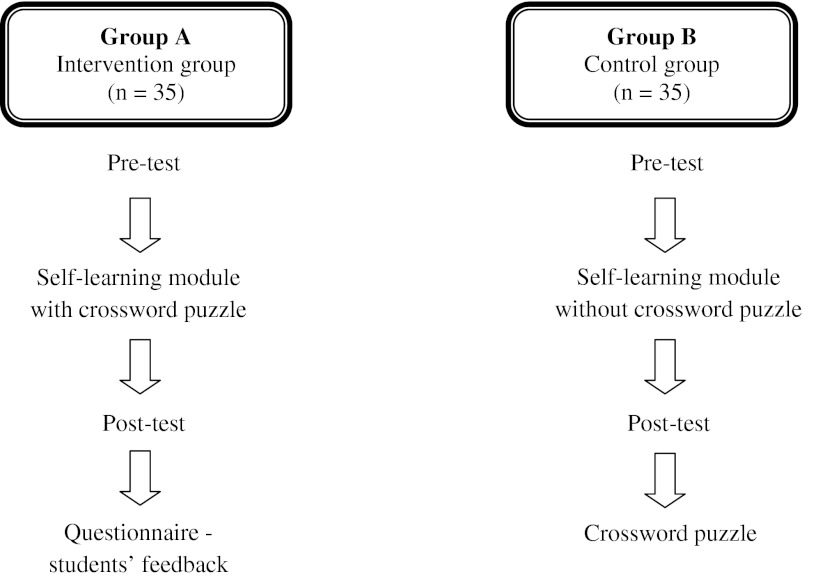
Schematic presentation of crossword puzzle activity

Group B (Control group) underwent the self-learning module without the crossword puzzles. In both groups, pre- and post-test assessment was done. As neither of the groups in the study should miss out on this innovation in medical education, Group B students (Control group) were able to do the crossword puzzles after the post-test. Students’ perceptions about crossword puzzles as a self-learning tool in pharmacology were only recorded for the intervention group, using a pre-validated questionnaire as described above (Fig. 2).

The crosswords for the two modules (antihypertensive drugs and antiepileptic drugs) were taken on two different occasions.

## Data analysis and statistics

Quantitative and qualitative data analysis was carried out. Quantitative data analysis comprised crossword completion index, pre- and post-test score comparison, learning effectiveness index, intervention effectiveness, students’ feedback on a 5-point Likert scale and crossword usefulness on a 10-point scale.

The crossword completion index was defined as the percentage of correctly solved clues. Student’s paired t-test was used to compare pre- and post-test scores. Post-test scores between the two groups were compared using the unpaired Student’s *t*-test. A *p* < 0.05 was considered significant.

The learning effectiveness index was calculated as absolute learning gain (%Post-test score − %Pre-test score) and relative learning gain (%Post-test score − %Pre-test/%Pre-test score). Class-average normalized gain measures the ratio of whole group performance to the maximum achievable improvement and is used by many educators as a measure for course effectiveness. Hence, in our study, effectiveness of intervention was evaluated by class-average normalized gain [g = (%Post-test score − %Pre-test score) − (100 − %Pre-test score)] [14]. A class-average normalized gain (g) of 0.3, i.e. 30 % was considered significant [14, 15].

Students’ responses to items 1–8, recorded on a 5-point Likert scale questionnaire, were expressed as percentages. Students’ responses to item 9 in the questionnaire (10-point scale for usefulness) were categorized as follows: 1–2 = not useful; 3–4 = slightly useful; 5–6 = moderately useful; 7–8 = useful; 9–10 = very useful. Comments in response to item 10 were evaluated qualitatively.

## Results

The compliance to the crossword puzzle activity in the students of the intervention group (Group A) was 100 % for both modules. The average crossword completion index was 92.86 %.

All students in the two groups took the pre-test as well as the post-test. The average test score in Group A improved significantly from 30.45 % (pre-test score: 6.09/20 ± 1.30) to 64.35 % (post-test score: 12.87/20 ± 1.39) (*p* < 0.05). The absolute learning gain was 33.9 % whereas the relative learning gain was 111.33 %. Group B also showed improvement in test scores from 30.15 % (pre-test score: 6.03/20 ± 1.39) to 48.70 % (post-test score: 9.74/20 ± 3.17). The absolute and relative gain in Group B was 18.55 and 61.53 %, respectively. A significant improvement in the post-test score of Group A was seen as compared with Group B (*p* < 0.001). Class-average normalized gain in Group A was 0.4872 (48.72 %) whereas in Group B it was 0.2656 (26.56 %) (Table 1).

**Table 1:** Pre-test scores, post-test scores and learning gain

Groups	Pre-test score	Post-test score	Absolute learning gain	Relative learning gain	Class-average normalized gain
A (n = 35)	6.09 ± 1.30 (30.45)	12.87 ± 1.39*^,@^ (64.35)	33.9	111.33	48.72
B (n = 35)	6.03 ± 1.39 (30.15)	9.74 ± 3.17* (48.70)	18.55	61.53	26.56

Pre- and post-test scores are expressed as mean ± SD (percentage). Values of absolute gain, relative gain, class-average normalized gain are expressed in percentages

*p < 0.05 using paired t-test (pre-test score vs post-test score for both groups)

^@^ *p* < 0.001 using unpaired t-test (Group A post-test score vs Group B post-test score)

The response rate for the questionnaire was 100 %. The Cronbach’s alpha, a measure for internal consistency of questionnaire items, was 0.825.

The students enjoyed doing the crossword puzzles. Of the students, 82.86 % strongly agreed on this point, 71.43 % students were of the strong opinion that their knowledge about antihypertensive and antiepileptic drugs was enhanced as a result of the crossword activity and 65.71 % strongly felt that the crosswords were helpful to remember and recall the drug names and promoted active learning as well.

Of the students, 60 % felt that the crossword puzzle activity was challenging and problem solving, while 68.57 % students were of the opinion that the crossword puzzle clues emphasized the core area of the topic.

Overall, 85.71 % students strongly felt that the crossword puzzle was useful as a self-learning tool and enhanced learning through recreation, and 77.14 % students strongly agreed that crossword puzzles should be incorporated in the pharmacology curriculum as a self-learning tool (Table 2).

**Table 2:** Students’ perceptions about crossword puzzles as a self-learning tool

Sr. No.	Statements	SD	D	N	A	SA
1	Enjoyable experience to solve crossword puzzle	0 (0.00)	0 (0.00)	0 (0.00)	6 (17.14)	29 (82.86)
2	Enhanced knowledge of antihypertensive and antiepileptic drugs	0 (0.00)	0 (0.00)	3 (8.57)	7 (20.00)	25 (71.43)
3	Helped to remember drug names	0 (0.00)	0 (0.00)	5 (14.29)	7 (20.00)	23 (65.71)
4	Challenging and problem solving	0 (0.00)	0 (0.00)	1 (2.86)	13 (37.14)	21 (60.00)
5	Enhances learning through recreation	0 (0.00)	0 (0.00)	0 (0.00)	5 (14.29)	30 (85.71)
6	Promotes active learning	0 (0.00)	0 (0.00)	0 (0.00)	12 (34.29)	23 (65.71)
7	Emphasizes core area of topic	0 (0.00)	0 (0.00)	1 (2.86)	10 (28.57)	24 (68.57)
8	Incorporation in pharmacology curriculum as self-learning tool/small group discussion	0 (0.00)	0 (0.00)	3 (8.57)	5 (14.29)	27 (77.14)

Values in parentheses indicate percentages. Responses recorded on 5-point Likert Scale: *SD* strongly disagree, *D* disagree, *N* neutral, *A* agree, *SA* strongly agree

Responses recorded on a 10-point rating scale for usefulness were as follows: 7 = 11.43 %, 8 = 48.57 %, 9 = 22.86 %, and 10 = 17.14 %. Thus, according to categorization of scores (described under "Materials and methods" section), 60 % of students found this intervention useful whereas 40 % perceived its educational value as very useful.

Free comments in response to item 10 in the questionnaire were analyzed and categorized as strengths and suggestions (Table 3).

**Table 3:** Strengths and suggestions for crossword puzzle activity

Strengths	Suggestions
Recreational method of learning in small group	*‘Students should be pre*-*informed’*.
*‘Helps to remember MCQ points’*.	Test in the form of crossword puzzle should be taken in class after completion of topic
*‘Helps to categorize drug names according to different functions’*.	Topic-wise crossword activity should be taken throughout the term
Crossword puzzle was challenging	Tutorials should be based on crossword puzzles
Crossword puzzles helpful in revision	Should be conducted in exam going term to facilitate learning without fear

## Discussion

The majority of Indian medical schools follow a discipline-based curriculum. Pharmacology is a fact-filled subject in the second professional MBBS course in the Indian medical curriculum. Students often find it difficult to understand concepts in pharmacology when they move from the first to the second professional level (II MBBS). Problems in recalling drug names during viva-voce of pharmacology are a routine observation by faculties. McDonald and Saarti [16] also mentioned that learning a fact-filled subject such as pharmacology should be less of a chore and more of a pleasurable experience. Active learning strategies in pharmacology may overcome these problems. Various educational researchers have advocated incorporation of active learning strategies into the curriculum to improve understanding and learning [3–5, 10, 11, 17]. Self-directed learning promotes active learning and develops critical thinking. We used crossword puzzles in pharmacology as a self-learning tool to promote active learning. The objectives of this study were to evaluate their usefulness and students’ perception towards this innovation.

This study concludes that in pharmacology the crossword puzzle is a useful self-learning tool. Effectiveness of this intervention was evident from improved post-test scores, absolute and relative learning gain and class-average normalized gain and students’ acceptability towards this innovation.

We evaluated the effectiveness of crossword puzzles in pharmacology using pre-test, post-test and learning gain. Although an improvement in the post-test score was observed in both groups, absolute as well as relative learning gain was observed to be greater in the intervention group as compared with the control group. In addition, class-average normalized gain in the intervention group was more than 0.3 (30 %).

Class-average normalized gain (g) has been used as a measure of effectiveness of an educational intervention by various researchers [18]. Hake (2002) stated that class-average normalized gain offers a comparative measure for course effectiveness over diverse student populations with widely varying average pre-test scores. Thus, it diminishes the confounding effect of pre-course knowledge [19]. Hence, we used class-average normalized gain (g) as a measure of effectiveness for our educational intervention, in addition to learning gain. Hake categorized a class-average normalized gain of 0.1–0.29 as low gain (g), 0.3–0.69 as medium gain and 0.7–1.0 as high gain [15, 18]. In our study, we observed the class-average normalized gain (g) of 0.4872 (48.72 %) in the intervention group as compared with 0.2656 (26.56 %) in control group. That means that the intervention group had an average gain of 48.72 % of the maximum possible average gain. Thus, according to Hake’s criteria, the crossword puzzle in pharmacology was a moderately effective educational intervention in the form of a self-learning tool.

A literature search showed that none of the studies in medical education used a randomized control design and pre-test/post-test analysis as measure to determine effectiveness of crossword puzzles. Hence, no previous data are available for comparison. However, a randomized control design and pre-test/post-test analysis was used to evaluate the crossword puzzle as a tool to improve the vocabulary mastery of eighth grade students at a high school in Riau, which showed improved vocabulary in the intervention group as compared with the control group [20].

Pre-test/post-test measurement in an educational intervention is often associated with extraneous variables such as history (any event, other than the planned treatment event that occurs between the pre- and post-test measurement and has an influence on the dependent variable); testing (any change on the second administration of a test as a result of having previously taken the test); differential attrition (differential loss of participants from the various comparison groups); Hawthorne effect (knowing that he/she is being tested may affect the results) and halo effect (human tendency to respond positively or negatively to a situation) [14, 21]. These extraneous variables are threats for the internal validity of results, and results of educational research involving evaluation of knowledge gain out of educational intervention may be affected. However, the threats such as history, testing, Hawthorne effect and halo effect do not affect two-group or multi-group designs. In our study, we used a two-group design. Pre-test, post-test and self-learning modules with intervention were administered on the same day. Significant gains in the post-test score would not be possible without intervention in this type of short duration course module. In addition, the randomized controlled design of our study further reduces the risk of such threats. Hence, we argue that many of these threats did not influence our educational intervention.

Effectiveness of the crossword puzzle as a self-learning tool is sustainable due to the positive feedback received from the students. Students’ feedback indicated that they enjoyed this new teaching–learning activity. Thorndike’s law of effect states that responses that are made just prior to pleasant events are more likely to be repeated and learnt. The crossword puzzle activity was a pleasurable experience for the students. They perceived crossword puzzles as a challenging, problem solving, useful, and recreational tool for learning. Hence, we can argue that retention was better in the intervention group as compared with the control group. As far as students’ perceptions towards this tool are concerned, our findings were consistent with previous studies [3, 11, 22]. Students have suggested that they should have been pre-informed about the crossword puzzle activity. Nevertheless, we deliberately used this strategy of not pre-informing to avoid any confounding effect of pre-knowledge on pre-test/post-test analysis. A few students felt that the crossword puzzles used in this study were less problem solving and challenging as evident from their suggestion to use more extensive clues. This is useful feedback for us to prepare crossword puzzles with different difficulty levels.

Crossword puzzles have a unique feature of self-correcting due to the length of each word and overlap of each answer from the other answer [11]. This unique feature helps students to correct their mistakes instantaneously. This develops their critical thinking and helps them retain the knowledge gained. Bailey et al. [10] developed educational puzzles as a supplementary tool to promote learning, enhance problem-solving skills to complement the information provided through traditional teaching. They also found crossword puzzles a useful and fun learning tool to evaluate their own knowledge. Saxena et al. [3] described crossword puzzles as useful for assessing near transfer, which is called for when students encounter problems very similar to the problems they worked on during the learning stage. In our study, students’ performance improved after the intervention. Hence, we can claim the crossword puzzle as a useful inventory of active learning methods.

The important parameters considered for successful implementation of an educational intervention are its utility (effectiveness), acceptability and feasibility [23]. The crossword puzzle in pharmacology as a self-learning tool was well perceived by the students. Crossword puzzles were helpful for revising learnt topics, for remembering and recalling drug names and were perceived as a recreational method of learning in a non-threatening environment by the students. These strengths indicate students’ acceptability of this self-learning tool.

Although preparing topic-specific crossword puzzles is somewhat time-consuming, the advantage is that this intervention is easy to implement. Tutorials and small group teaching can be conducted using this intervention. Crossword puzzle activities are easy to implement either using hard copies (as described in the study) or electronically. A range of software is available on the internet to prepare an electronic form of crossword puzzle. Moderate effectiveness of crossword puzzles and their acceptability by students favour their usefulness as a recreational self-learning tool in pharmacology.

## Conclusion

Crossword puzzles in pharmacology were found to be a useful self-learning tool. Test scores improved when crossword puzzles, designed to improve the retention of drug names, were incorporated in self-study modules. Students rated this activity highly. Students perceived the crossword puzzles as a challenging and effective self-learning tool. Students’ acceptance of the crossword puzzles further favours their usefulness as a self-learning tool. The crossword puzzle is an effective tool for recreational learning and can be used as an active learning strategy to promote self-directed learning.

## Essentials

- Post-test score, absolute learning gain and relative learning gain were better in the students in the intervention group as compared with the control group students.
- Class-average normalized gain in the intervention group was 48.72 %, which is more than 30 %, indicating moderate effectiveness of crossword puzzles as an educational intervention.
- Crossword puzzles are challenging, problem solving and effective in facilitating active learning and enhancing students’ knowledge.
- Students’ acceptance of crossword puzzles as a self-learning and recreational tool for learning favours their usefulness.
